# Rosiglitazone promotes ENaC-mediated alveolar fluid clearance in acute lung injury through the PPARγ/SGK1 signaling pathway

**DOI:** 10.1186/s11658-019-0154-0

**Published:** 2019-05-28

**Authors:** Jing He, Di Qi, Xu-mao Tang, Wang Deng, Xin-yu Deng, Yan Zhao, Dao-xin Wang

**Affiliations:** Department of Respiratory Medicine, The Second Affiliated Hospital of Chongqing Medical University, 76 Linjiang Road, Yuzhong District, Chongqing, 400010 China

**Keywords:** Acute lung injury, Alveolar epithelial sodium channel, Alveolar fluid clearance, Peroxisome proliferator-activated receptor γ, Serum and glucocorticoid induced kinase-1

## Abstract

**Background:**

Pulmonary edema is one of the pathological characteristics of acute respiratory distress syndrome (ARDS). The epithelial sodium channel (ENaC) is thought to be the rate-limiting factor for alveolar fluid clearance (AFC) during pulmonary edema. The peroxisome proliferator-activated receptor γ (PPARγ) agonist rosiglitazone was shown to stimulate ENaC-mediated salt absorption in the kidney. However, its role in the lung remains unclear. Here, we investigated the role of the PPARγ agonist in the lung to find out whether it can regulate AFC during acute lung injury (ALI). We also attempted to elucidate the mechanism for this.

**Methods:**

Our ALI model was established through intratracheal instillation of lipopolysaccharide (LPS) in C57BL/6 J mice. The mice were randomly divided into 4 groups of 10. The control group underwent a sham operation and received an equal quantity of saline. The three experimental groups underwent intratracheal instillation of 5 mg/kg LPS, followed by intraperitoneal injection of 4 mg/kg rosiglitazone, 4 mg/kg rosiglitazone plus 1 mg/kg GW9662, or only equal quantity of saline. The histological morphology of the lung, the levels of TNF-α and IL-1β in the bronchoalveolar lavage fluid (BALF), the level of AFC, and the expressions of αENaC and serum and glucocorticoid-induced kinase-1 (SGK1) were determined. Type 2 alveolar (AT II) cells were incubated with rosiglitazone (15 μM) with or without GW9662 (10 μM). The expressions of αENaC and SGK1 were determined 24 h later.

**Results:**

A mouse model of ALI was successfully established. Rosiglitazone significantly ameliorated the lung injury, decreasing the TNF-α and IL-1β levels in the BALF, enhancing AFC, and promoting the expressions of αENaC and SGK1 in ALI mice, which were abolished by the specific PPARγ blocker GW9662. In vitro, rosiglitazone increased the expressions of αENaC and SGK1. This increase was prevented by GW9662.

**Conclusions:**

Rosiglitazone ameliorated the lung injury and promoted ENaC-mediated AFC via a PPARγ/SGK1-dependent signaling pathway, alleviating pulmonary edema in a mouse model of ALI.

**Electronic supplementary material:**

The online version of this article (10.1186/s11658-019-0154-0) contains supplementary material, which is available to authorized users.

## Background

Acute respiratory distress syndrome (ARDS) is a severe illness that is characterized by diffuse alveolar damage, increased lung permeability and pulmonary edema [1]. Pulmonary edema is induced when inflammation causes damage to alveolar epithelial and endothelial cells, and results in alveolar fluid buildup and stubborn hypoxemia. Any delay to the resolution of pulmonary edema prevents the recovery of effective gas exchange and oxygenation of the lung [2, 3]. Therefore, timely clearance of alveolar fluid from the edema is of great importance to ARDS patients.

Epithelial sodium channel (ENaC) is a multimeric protein that plays a critical role in the process of alveolar fluid clearance (AFC). Consisting of at least three subunits, ENaC is located in the apical membrane of alveolar epithelial cells. Its α subunit is necessary to form functional ENaC, while the β and γ subunits promote the channel’s activity [4–6].

Reabsorption of pulmonary edema starts when sodium enters the alveolar epithelial cells through ENaC. It is subsequently pumped out by Na^+^-K^+^-adenosine triphosphatase (Na^+^-K^+^-ATPase) at the basal membrane. The osmotic pressure caused by Na^+^ transport drives water reabsorption [7]. Therefore, ENaC is thought to be the rate-limiting factor for AFC during pulmonary edema.

Serum and glucocorticoid inducible kinase 1 (SGK1) is a member of the AGC kinase family [8]. In mammals, ubiquitously expressed SGK1 participates in the regulation of cell proliferation [9], hormone release [10], apoptosis [11] and ion transport [12]. Our previous studies have demonstrated that SGK1 is of great importance in the regulation of ENaC-mediated AFC during acute lung injury [13]. However, aspects of SGK1 regulation remain unclear.

Peroxisome proliferator-activated receptors (PPARs) are ligand-activated transcription factors belonging to a nuclear hormone receptor superfamily containing three isoforms: α, β/δ, and γ [14]. PPARγ is expressed primarily in adipose tissue, where it plays a critical role in adipocyte differentiation and lipid metabolism [15]. In addition, PPARγ has also been detected in other tissues, including the kidney and lungs [15]. In previous studies, PPARγ agonists were shown to stimulate ENaC-mediated salt absorption in the kidney [16, 17]. However, the biological role of PPARγ agonists in the lung remains unclear. Therefore, in this study, we investigated the role of the PPARγ agonist rosiglitazone in the lung to find out whether it can regulate AFC during acute lung injury. We also attempted to elucidate its mechanism.

## Materials and methods

### Animals

Eight-week old male C57BL/6 J mice weighing 22–25 g were purchased from the Laboratory Animal Center of Chongqing Medical University and housed under specific pathogen-free conditions in a temperature- and humidity-controlled environment with a 12/12 h day/night cycle. The mice were allowed food and water ad libitum. All surgeries were performed under sodium pentobarbital anesthesia, and all efforts were made to minimize suffering. All animal procedures were approved by the Ethics Committee of Animal Experiments of the Second Affiliated Hospital of Chongqing Medical University. This study was performed in strict accordance with *The Guide for the Care and Use of Laboratory Animals* (Eighth Edition, 2011, published by The National Academies Press, USA).

### Main reagents

Lipopolysaccharide (LPS, *Escherichia coli* serotype 055:B5), sodium pentobarbital, Evans blue dye, collagenase and trypsin were purchased from Sigma. ELISA kits were purchased from Abcam. Rosiglitazone (RGZ, C_18_H_19_N_3_O_3_S, purity ≥98%) and GW9662 (C_13_H_9_C_l_N_2_O_3_, purity ≥95%) were purchased from Santa Cruz Biotechnology. Anti-αENaC antibody, anti-SGK1 antibody, anti-pSGK1 (Ser422) antibody, anti-GAPDH antibody, and all secondary antibodies were all purchased from Abcam. RNAiso plus, PrimeScript RT Reagent Kit (Perfect Real Time), and SYBR Premix Ex Taq II were all purchased from TaKaRa Biotechnology.

### Animal experimental protocol

Mice were randomly divided into 4 groups of 10: control, LPS, RGZ (LPS + rosiglitazone), and GW (GW9662 + LPS + rosiglitazone). The mice were all anesthetized with 50 mg/kg sodium pentobarbital by intraperitoneal injection. The three experimental groups received 5 mg/kg LPS in 50 μl of sterile saline, which was instilled intratracheally with an indwelling vein needle. The control group only received 50 μl sterile saline. Afterwards, the GW group received an intraperitoneal injection of 1 mg/kg GW9662. Thirty minutes later, the RGZ group and the GW group received an intraperitoneal injection of 4 mg/kg rosiglitazone in 100 μl saline while the other groups were injected with the same volume of saline.

After resuscitation, the mice were housed as previously mentioned. The animals were killed after 24 h and their lungs were removed for the next experiments. Lungs from 5 mice from each group were used to measure the alveolar fluid clearance. For the other 5 mice from each group, the right lungs were used for lung histology, the left upper lungs were used for real-time PCR, and the left lower lungs were used for western blot after whole lung bronchoalveolar fluid lavage (BALF).

### Cell isolation, culture and intervention

Type 2 alveolar (AT II) cells were isolated from C57BL/6 J mice via collagenase and trypsin digestion of lung tissue and purified by adherence to IgG-coated plates, as described by Dobbs et al. [18]. Cell viability was assessed with trypan blue staining and the identity of the cells was determined via immunocytochemical detection of surfactant protein C, which is indicative of AT II cells.

AT II cells were seeded onto plastic culture dishes and cultured with DMEM/F12 containing 10% fetal bovine serum (FBS), 100 U/ml penicillin and 0.1 mg/ml streptomycin in a 37 °C incubator containing 5% CO_2_. On the second day, the interventions were administered. The control group received an equal volume of sterile phosphate-buffered saline (PBS). The RGZ group received 15 μM rosiglitazone and an equal volume of sterile PBS. The GW group received 10 μM GW9662 and 30 min later, 15 μM rosiglitazone. Twenty-four hours later, cells were collected and further experiments were performed. The doses of the drugs were determined based on previous research [17, 19] and our preliminary experiments (Additional file 1: Figures S1~S4).

### Evaluation of lung histology

The lungs were harvested and immediately fixed in 4% paraformaldehyde for 24 h. Then, they were embedded in paraffin, cut into sections, and stained with hematoxylin and eosin (H&E) for optical microscopy. A semi-quantitative scoring system was adopted to evaluate lung injury as previously described, with a scale of 0 to 4 points based on combined assessments of inflammatory cell infiltration, alveolar septa thickness, intra-alveolar and interstitial edema, and hemorrhage. A score of 0 represented no injury, 1 represented slight injury, 2 represented moderate injury, 3 represented severe injury, and 4 represented very severe injury [20].

### Alveolar fluid clearance

AFC determinations were done as previously described [21]. Briefly, after the lung was removed integrally, 1 ml warm saline containing Evans blue dye-labeled 5% albumin was injected into it. Then 2 ml oxygen was injected to distribute the saline evenly in the alveolar spaces. The lungs were incubated at 37 °C and inflated at an airway pressure of 7 cm H_2_O with oxygen for 1 h. AFC was calculated as follows:

$$ \mathrm{AFC}=\left[\left(\mathrm{Vi}-\mathrm{Vf}\right)/\mathrm{Vi}\right]\times 100\%\mathrm{Vf}=\left(\mathrm{Vi}\times \mathrm{Ei}\right)/\mathrm{Ef} $$

where V represents the volume of injected albumin solution (i) and final alveolar fluid (f), and E represents the injected (i) and final (f) concentration of Evans blue-labeled 5% albumin solution.

### TNF-α and IL-1β levels in bronchoalveolar fluid lavage

BALF was acquired using the established procedure [22]. Briefly, mice were anesthetized with sodium pentobarbital (50 mg/kg). Then the tracheas and lungs were exposed. A catheter was intubated into the trachea and bronchoalveolar lavage was performed with a 1 ml syringe through 3 cycles of instillation and aspiration with 1 ml warm saline every time. More than 90% of the BALF was collected from each mouse and centrifuged at 800 rpm for 10 min at 4 °C to remove cell debris. The supernatants were stored at − 80 °C for further research. Measurements of TNF-α (ab208348) and IL-1β (ab242234) were analyzed via enzyme-linked immunosorbent assay. The assay ranges of the two kits were 46.88–3000 pg/ml and 28.1–1800 pg/ml. The respective inter-assay CV values were 9.8 and 3.5%. The intra-assay CV values were 6.7 and 3.1%, respectively. All were used in accord with the manufacturers’ instructions.

### Real-time PCR analysis

Total RNA from the tissues and cells was extracted using RNAiso plus solution (TaKaRa). The concentration and purity of the RNA were estimated on a spectrophotometer. 1 μg of total RNA was used for synthesizing the cDNA using the PrimeScript RT Reagent Kit (Perfect Real Time). cDNA was used for real-time PCR using SYBR Premix Ex Taq II (Takara). All primers were synthesized by TaKaRa: αENaC (forward) 5′-TAC GCG ACA ACA ATC CCC AAG TGG-3′ and (reverse) 5′-ATG GAA GAC ATC CAG AGA TTG GAG-3′; SGK1 (forward) 5′-CGG AAT GTT CTG TTG AAG AAT GTG -3′, (reverse) 5′-TGT CAG CAG TCT GGA AAG AGA AGT -3′; and GAPDH (forward) 5′-CAA GGT CAT CCA TGA CAA CTT TG -3′, (reverse) 5′-GTC CAC CCT GTT GCT GTA G-3′. The PCR parameters were 95 °C for 30 s, followed by 40 cycles at 95 °C for 5 s and 60 °C for 30 s. The results were normalized to GAPDH as an internal control.

### Protein extraction and western blot analysis

Total proteins and membrane proteins were respectively obtained with Total Protein Extraction Kits and Membrane Protein Extraction Kits (KeyGEN BioTECH) according to the manufacturer’s instructions. The concentration of each protein sample was determined using a BCA protein assay kit (KeyGen BioTECH). The total proteins were used for the detection of SGK1 and pSGK1, while the membrane proteins were used for the detection of αENaC. An equal amount of protein (50 μg) from each sample was separated via electrophoresis on SDS-PAGE and transferred to polyvinylidene fluoride membranes. After blocking with 5% non-fat milk for 1 h, the membranes were incubated with anti-αENaC (1:800), anti-pSGK (Ser422) (1:1000), and anti-SGK (1:1000) primary antibodies overnight at 4 °C. GAPDH was used as a loading control. Then, the membranes were incubated with a secondary antibody (1:5000) at room temperature for 2 h. Using an enhanced chemiluminescence (ECL) method, protein bands were detected using a Bio-Rad Gel Imaging System and analyzed with Quantity One software (Bio-Rad).

### Statistical analysis

All data are presented as the means ± S.E.M. Data were analyzed using a one-way analysis of variance (ANOVA) followed by the least-square difference (LSD) post-test for multiple comparisons or Kruskal-Wallis H analysis using SPSS 13.0 software (SPSS Inc.). *p* < 0.05 was considered statistically significant.

## Results

### Rosiglitazone alleviated lung injury in LPS-induced ALI

**Fig. 1:**
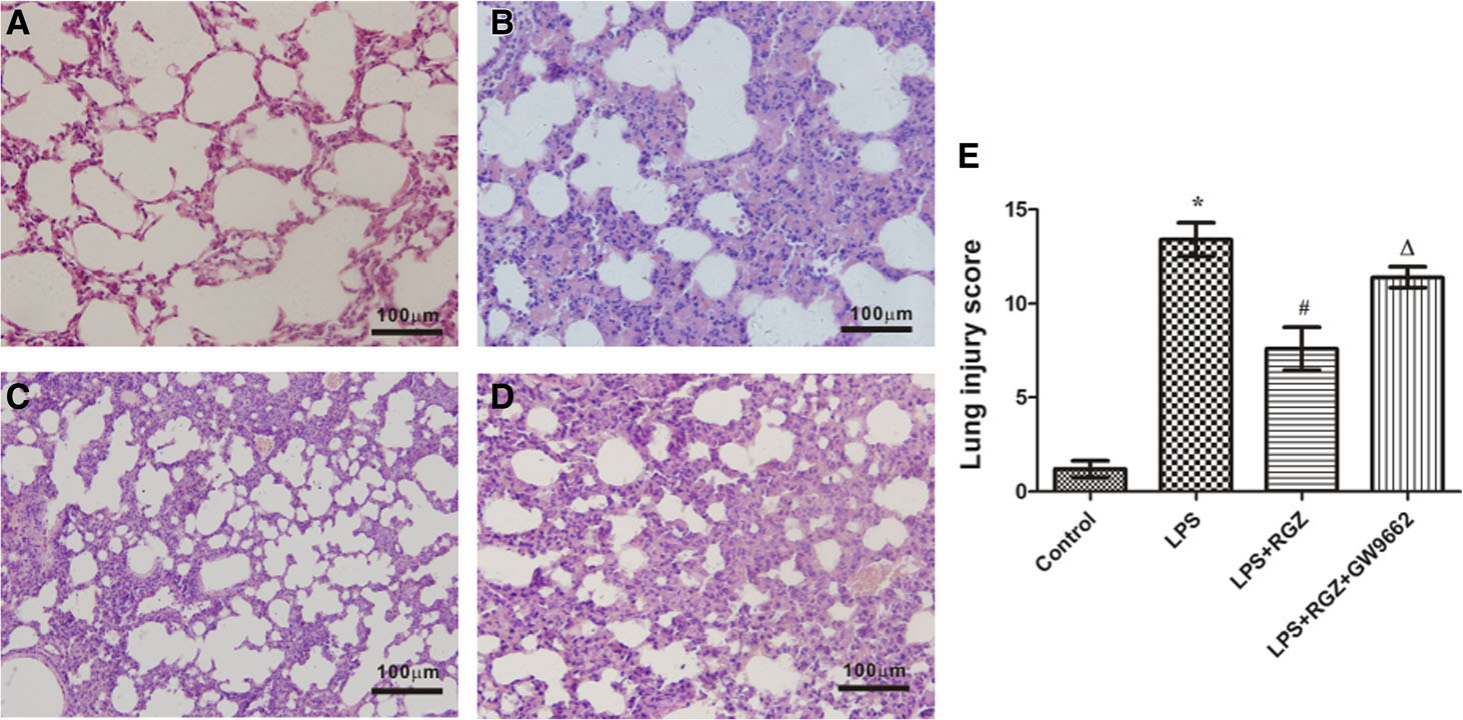
Effect of rosiglitazone on pulmonary morphology in mouse models of ALI. HE staining was used to determine the morphological changes to the lungs in mouse models of ALI. A representative figure from each group is shown. **a** Control group. **b** LPS group. **c** RGZ group (received LPS + rosiglitazone). **d** GW group (received LPS + rosiglitazone + GW9662). The lung injury score in each group (**e**) is shown as the mean ± SEM (*n* = 5). The data were analyzed using ANOVA followed by the LSD post-test for multiple comparisons with SPSS 13.0 software. *p < 0.05 vs. control group; ^#^*p* < 0.05 vs. LPS group; ^*Δ*^*p* < 0.05 vs. LPS + RGZ group

H&E staining was used to evaluate the pathological morphology of the mouse lungs and compared with the control group. We observed obvious destruction of alveolar structure, inflammatory cell infiltration, thickening of the alveolar septa, and alveolar edema in the LPS group (Fig. 1a and b). Rosiglitazone significantly alleviated the alveolar edema and partly relieved the inflammation, which was prevented by GW9662 (Fig. 1c and d).

### Rosiglitazone decreased the inflammatory mediators in the bronchoalveolar lavage fluid

**Fig. 2:**
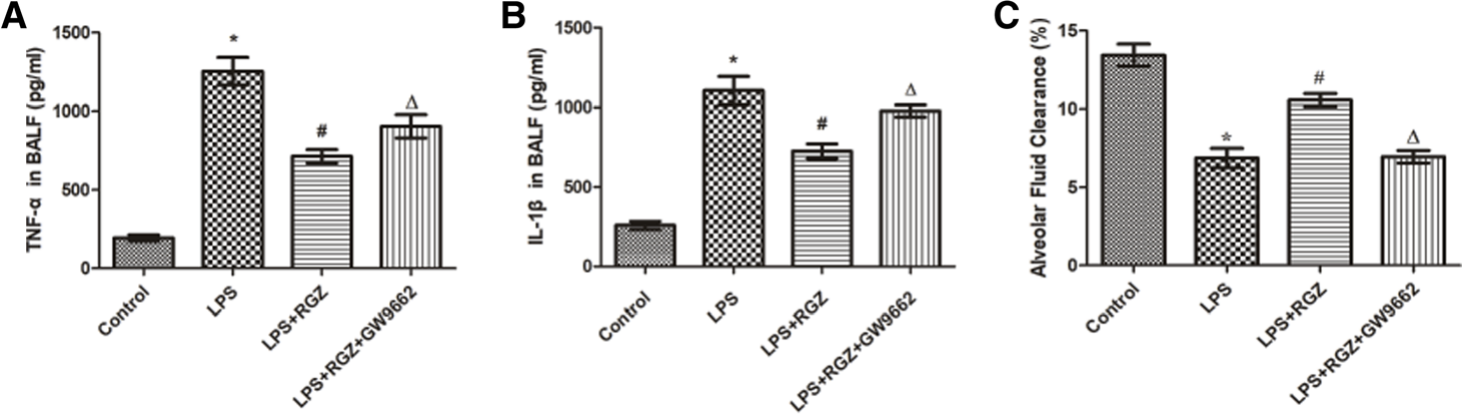
Effect of rosiglitazone on inflammatory mediators in broncho-alveolar lavage fluid (BALF) and alveolar fluid clearance (AFC) in mouse models of ALI. **a** and **b** The TNF-α (**a**) and IL-1β (**b**) levels in BALF were determined via ELISA. **c** The AFC in ALI mice was determined. The data are presented as the means ± SEM (*n* = 5) and analyzed using ANOVA followed by the LSD post-test for multiple comparisons with SPSS 13.0. *p < 0.05 vs. control group; ^#^*p* < 0.05 vs. LPS group; ^*Δ*^*p* < 0.05 vs. LPS + RGZ group

LPS caused inflammatory cascades in the lung, which promoted the production of a series of pro-inflammatory mediators, including TNF-α and IL-1β. In this study, LPS caused significant increases in TNF-α and IL-1β in the BALF compared with the control group (*p* < 0.05; Fig. 2a and b). Rosiglitazone decreased the levels of TNF-α and IL-1β in the BALF to a certain extent, but this decrease was prevented by GW9662 (*p* < 0.05; Fig. 2a and b).

### Rosiglitazone promoted alveolar fluid clearance in LPS- induced acute lung injury mouse model

In LPS-induced acute lung injury mouse, AFC was lower than in the control group (*p* < 0.05; Fig. 2c). Rosiglitazone alleviated the LPS-induced decrease in AFC. However, the effect of rosiglitazone was abolished by its inhibitor GW9662 (*p* < 0.05; Fig. 2c).

### Rosiglitazone increased the expressions of the SGK1, pSGK1 and αENaC in LPS-induced acute lung injury mouse model

**Fig. 3:**
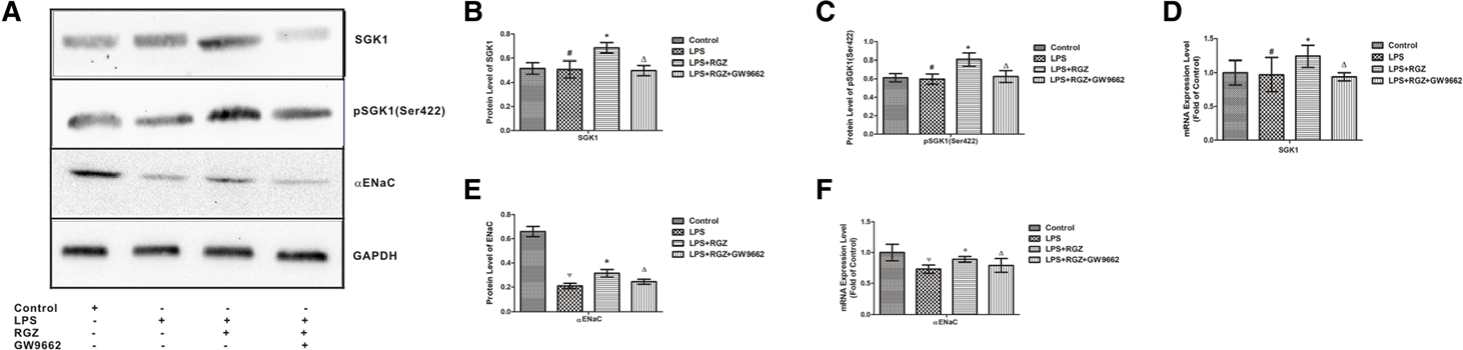
Effect of rosiglitazone on the expressions of αENaC and SGK1 in the lungs of ALI models. The protein expressions of SGK1 (**a** and **b**), pSGK1 (**a** and **c**), and αENaC (**a** and **e**) in ALI mouse models were examined via western blot analysis. The mRNA expressions of SGK1 (**d**) and αENaC (**f**) in ALI mouse models were examined using qPCR. The data are presented as the means ± SEM (n = 5) and analyzed using ANOVA followed by the LSD post-test for multiple comparisons with SPSS 13.0. ^#^*p* > 0.05 vs. control group; ^Ψ^*p* < 0.05 vs. control group; *p < 0.05 vs. LPS group; ^Δ^*p* < 0.05 vs. LPS + RGZ group

To investigate the mechanism accounting for the effect of rosiglitazone on AFC, we determined the expressions of SGK1, pSGK1 (Ser422) and membrane αENaC. Compared with the control group, LPS significantly decreased the mRNA and membrane protein expression levels of αENaC (*p* < 0.05; Fig. 3a, e and f), but not SGK1 (*p* > 0.05; Fig. 3a, b and d). Compared with the LPS group, rosiglitazone significantly increased both the mRNA and protein expression levels of SGK1, including the protein expression of pSGK1 (Ser422) (*p* < 0.05; Fig. 3a, b, c and d) and the mRNA and membrane protein expression levels of αENaC (*p* < 0.05; Fig. 3a, e and f) at the same time. However, the increases in pSGK1 (Ser422), SGK1 and αENaC were prevented by GW9662 (*p* < 0.05; Fig. 3a–f).

### Rosiglitazone increased the expression of the SGK1 and αENaC in AT II cells

**Fig. 4:**
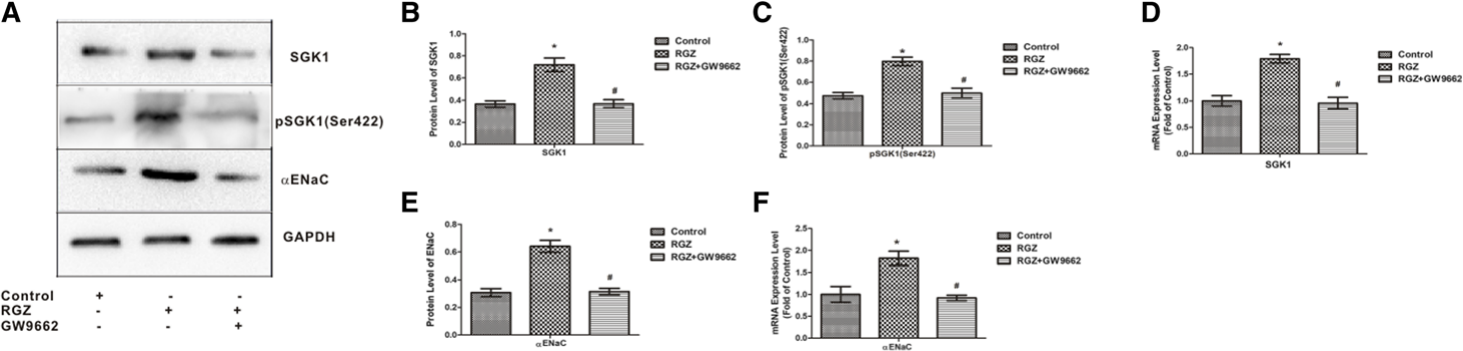
Effect of rosiglitazone on the expressions of αENaC and SGK1 in AT II cells. The in vitro protein expressions of SGK1 (**a** and **b**), pSGK1 (**a** and **c**), and αENaC (**a** and **e**) were examined via western blot. The in vitro mRNA expressions of SGK1 (**d**) and αENaC (**f**) were examined using qPCR. The data are presented as the means ± SEM (*n* = 5) and analyzed using ANOVA followed by the LSD post-test for multiple comparisons with SPSS 13.0. *p < 0.05 vs. control group; ^#^*p* > 0.05 vs.RGZ group

To further confirm the mechanism, isolated AT II cells were tested. In vitro, rosiglitazone increased the mRNA expression levels of SGK1 and αENaC, and enhanced the expressions of SGK1, pSGK1 (Ser422), and membrane αENaC. However, all the effects of rosiglitazone were inhibited by GW9662, which confirms the in vivo results (p < 0.05; Fig. 4a–f).

## Discussion

Our observations show an influence of the PPARγ agonist rosiglitazone on the AFC in ALI. Rosiglitazone treatment increased the total and phosphorylated expressions of SGK1, which was confirmed to have the capacity to upregulate the expression of ENaC on the cell surface.

Here, we built a model of ALI through intratracheal instillation of LPS. Successful establishment of the ALI model was confirmed by hallmarks present in the lung tissues such as neutrophil infiltration, alveolar septa thickening, and edema accumulation in the alveolar spaces.

Rosiglitazone has many advantages in treating patients with diabetes, including increasing insulin sensitivity, reducing blood glucose and hemoglobin A1c levels, inhibiting adipose tissue lipolysis hormones, and inhibiting inflammation [23, 24]. However, edema as a side effect restricts its clinical usage. Rosiglitazone prompts Na^+^ reabsorption in the kidney to induce sequential fluid retention [25, 26]. Interestingly, when pulmonary edema occurs, the situation is just the opposite. During pulmonary edema, it is of great importance to accelerate Na^+^ reabsorption to drive the alveolar fluid clearance. Whether rosiglitazone has any effect on alveolar fluid was unclear.

In our study, we found that rosiglitazone could alleviate pulmonary injury in ALI mice. We ascribed two reasons to this effect. Rosiglitazone was thought to have anti-inflammation capacity in previous research [27, 28]. Here, we also observed that rosiglitazone decreased the inflammatory mediator levels in the BALF, which concurred with the results of previous studies. On the other hand, rosiglitazone also promoted AFC, a mechanism independent of the alleviation of inflammation but dependent on ENaC. Rosiglitazone-stimulated expression of ENaC in ALI mice was significantly reduced by the PPARγ-specific blocker GW9662, indicating the effect was mediated by the PPARγ signaling pathway. In vitro, we also found that rosiglitazone increased the expression of ENaC in alveolar epithelial cells. However, GW9662 almost abolished the effect of rosiglitazone, indicating that PPARγ is an essential point in this signaling pathway. At the same time, we found a positive correlation between the expressions of SGK1 (pSGK1) and ENaC, indicating SGK1 was involved in this regulating process. Therefore, we further investigated the relationship between rosiglitazone, SGK1 (pSGK1) and ENaC.

SGK1 belongs to a subfamily of S/T kinases known as the AGC protein kinases. SGK1 is a functional convergence of various cell signaling pathways and phosphorylation cascades, playing important roles in ion channels, the cellular stress response, and cell survival. Our previous research revealed that SGK1 is an important signaling molecule participating in ion transport in alveolar epithelia. Activated SGK1 (pSGK1 at Ser422) can phosphorylate neural precursor cell-expressed developmentally downregulated protein 4–2 (Nedd4–2), a negative regulator of ENaC. pSGK1 phosphorylates Nedd4–2 to promote the interactions of Nedd4–2 with chaperonin 14–3-3 proteins instead of with ENaC, leading to the inhibition of ENaC ubiquitylation and further degradation. Thus, the number of ENaC remaining on the alveolar epithelial cell surface increases [29–31]. SGK1 was found to hold an anti-inflammatory function by phosphorylating TGF-β-activated kinase 1 (TAK1) [32]. This is another mechanism accounting for how rosiglitazone alleviates inflammation.

As a transcription factor, PPARγ controls downstream gene expression. PPARγ binds to PPAR response elements (PPRE) of target genes and forms heterodimerization with the retinoid X receptor (RXR) to trans-activate or trans-repress the goal gene through DNA-dependent or DNA-independent mechanisms [33]. Previous studies demonstrated that PPAR activators negatively interfere with the nuclear factor-κB (NF-κB), STAT and AP-1 signaling pathways to inhibit the activation of inflammatory response genes [27, 28, 34]. We also observed that rosiglitazone decreased the levels of inflammatory mediators in BALF, confirming this conclusion.

Meanwhile, rosiglitazone upregulated the expression of SGK1. Through bioinformatic analysis, Hong et al. found that in CCD cells, the SGK1 had PPRE located in the promoter site. Therefore, PPARγ could bind to the PPRE of SGK1 and heterodimerize with RXR to activate SGK1 gene transcription [35].

In this study, we found that the PPARγ agonist rosiglitazone increased the SGK1 expression from the transcription level, and accordingly the activated SGK1, which further upregulated ENaC expression and ENaC-mediated AFC in the lung.

However, there still is controversy about the mechanism of the PPARγ agonist rosiglitazone on Na^+^ transport. Renauld et al. [17] found the PPARγ agonist rosiglitazone increases ENaC expression at the plasma membrane of *Xenopus laevis* oocytes. Fu et al. [36] concluded from their research that the PPARγ agonist rosiglitazone promoted ENaC-mediated Na^+^ reabsorption in the connecting tubule cells. By contrast, Wilson et al. [37] concluded that the PPARγ agonist had no discernible effect on transepithelial Na^+^ absorption in H441 human distal airway epithelial cells and mouse renal collecting duct mpkCCD cells. The two opinions above are supported by different studies. In our current study, our data are consistent with the data of Renauld et al. [17] and Fu et al. [36], but are inconsistent with the data of Wilson et al. [37]. It is possible that the discrepancy may be attributed to different cell types, varied cellular environments and statuses, or different responses to diverse stimulants.

Our results show that rosiglitazone relieves lung injury. In a mouse model of ALI, it was found to stimulate ENaC-mediated AFC through the PPARγ/SGK1 signaling pathway to mitigate pulmonary edema. In addition, our results suggest a mechanical basis for the control of ENaC-mediated AFC by rosiglitazone, which may facilitate the development of novel related therapies for pulmonary edema. However, further work is still needed to test the effects of rosiglitazone on large mammal models of ALI and humans with ALI.

## Conclusion

The PPARγ agonist rosiglitazone stimulates ENaC-mediated AFC through the PPARγ/SGK1 signaling pathway to mitigate pulmonary edema in a mouse model of ALI. This study may indicate a direction for future study on a therapeutic target for pulmonary edema in ARDS/ALI.

## Additional file

Additional file 1: **Figure S1.** The effect of different doses of rosiglitazone on the expression of *αENaC* mRNA in mouse models of acute lung injury (ALI). The expression of *αENaC* mRNA increased gradually with increasing doses of rosiglitazone up to 4 mg/kg, which significantly increased *αENaC* mRNA expression. A further increase of rosiglitazone (8 mg/kg) did not further increase the expression of *αENaC* mRNA. The data are presented as the means ± SEM (*n* = 3) and analyzed with SPSS 13.0using ANOVA followed by LSD post-test for multiple comparisons. ^#^*p* > 0.05 vs. control group; *p < 0.05 vs. control group; ^Δ^*p* > 0.05 vs. RGZ 4 mg/kg group. **Figure S2.** The effect of different doses of GW9662 on the rosiglitazone-increased expression of *αENaC* mRNA in mouse models of ALI. The expression of *αENaC* mRNA decreased gradually with increasing doses of GW9662 up to 1 mg/kg, which significantly decreased *αENaC* mRNA expression compared with the control group. A further increase of GW9662 (2 mg/kg) did not further decrease the expression of *αENaC* mRNA. The data are presented as the means ± SEM (*n* = 3) and analyzed with SPSS 13.0using ANOVA followed by LSD post-test for multiple comparisons. ^#^*p* > 0.05 vs. control group; *p < 0.05 vs. control group; ^Δ^*p* > 0.05 vs. RGZ 4 mg/kg + GW9662 1 mg/kg group. **Figure S3.** The effect of different doses of rosiglitazone on the expression of *αENaC* mRNA in alveolar epithelial cells. The expression of *αENaC* mRNA in alveolar cells increased gradually with increasing doses of rosiglitazone, up to 15 μM, which significantly increased the *αENaC* mRNA expression in alveolar epithelial cells. A further increase of rosiglitazone (20 μM) did not further increase the expression of *αENaC* mRNA. The data are presented as the means ± SEM (*n* = 3) and analyzed with SPSS 13.0 using ANOVA followed by LSD post-test for multiple comparisons. ^#^*p* > 0.05 vs. control group; *p < 0.05 vs. control group; ^Δ^*p* > 0.05 vs. RGZ 15 μM group. **Figure S4.** The effect of different doses of GW9662 on the rosiglitazone-increased expression of *αENaC* mRNA in alveolar epithelial cells. The expression of *αENaC* mRNA decreased gradually when increasing the dose of GW9662 in alveolar epithelial cells up to 10 μM GW9662, which significantly decreased the *αENaC* mRNA expression compared with the control group. A further increase of GW9662 (20 μM) did not further decrease the expression of *αENaC mRNA*. The data are presented as the means ± SEM (n = 3) and analyzed with SPSS 13.0 using ANOVA followed by LSD post-test for multiple comparisons. ^#^*p* > 0.05 vs. control group; *p < 0.05 vs. control group; ^Δ^*p* > 0.05 vs. RGZ 15 μM + GW9662 10 μM group. (ZIP 760 kb)

## Abbreviations

AFC: alveolar fluid clearance
ALI: acute lung injury
ARDS: acute respiratory distress syndrome
AT II: type II alveolar epithelial cell
ENaC: epithelial sodium channel
LPS: lipopolysaccharide
Nedd4–2: neural precursor cell-expressed developmentally downregulated protein 4–2
PPARs: peroxisome proliferator-activated receptors
RGZ: rosiglitazone
SGK1: serum and glucocorticoid-inducible kinase 1
