# Supplementary material for: Rosiglitazone promotes ENaC-mediated alveolar fluid clearance in acute lung injury through the PPARγ/SGK1 signaling pathway
Source: Cell Mol Biol Lett. 2019 May 28;24:35. doi: 10.1186/s11658-019-0154-0 (PMC6540532; doi:10.1186/s11658-019-0154-0)
Supplement: Supplementary file 1 — Figure S1. The effect of different doses of rosiglitazone on the expression of αENaC mRNA in mouse models of acute lung injury (ALI). The expression of αENaC mRNA increased gradually with increasing doses of rosiglitazone up to 4 mg/kg, which significantly increased αENaC mRNA expression. A further increase of rosiglitazone (8 mg/kg) did not further increase the expression of αENaC mRNA. The data are presented as the means ± SEM (n = 3) and analyzed with SPSS 13.0using ANOVA followed by LSD post-test for multiple comparisons. #p > 0.05 vs. control group; *p < 0.05 vs. control group; Δp > 0.05 vs. RGZ 4 mg/kg group. Figure S2. The effect of different doses of GW9662 on the rosiglitazone-increased expression of αENaC mRNA in mouse models of ALI. The expression of αENaC mRNA decreased gradually with increasing doses of GW9662 up to 1 mg/kg, which significantly decreased αENaC mRNA expression compared with the control group. A further increase of GW9662 (2 mg/kg) did not further decrease the expression of αENaC mRNA. The data are presented as the means ± SEM (n = 3) and analyzed with SPSS 13.0using ANOVA followed by LSD post-test for multiple comparisons. #p > 0.05 vs. control group; *p < 0.05 vs. control group; Δp > 0.05 vs. RGZ 4 mg/kg + GW9662 1 mg/kg group. Figure S3. The effect of different doses of rosiglitazone on the expression of αENaC mRNA in alveolar epithelial cells. The expression of αENaC mRNA in alveolar cells increased gradually with increasing doses of rosiglitazone, up to 15 μM, which significantly increased the αENaC mRNA expression in alveolar epithelial cells. A further increase of rosiglitazone (20 μM) did not further increase the expression of αENaC mRNA. The data are presented as the means ± SEM (n = 3) and analyzed with SPSS 13.0 using ANOVA followed by LSD post-test for multiple comparisons. #p > 0.05 vs. control group; *p < 0.05 vs. control group; Δp > 0.05 vs. RGZ 15 μM group. Figure S4. The effect of different doses of GW9662 on the rosiglit [file 11658_2019_154_MOESM1_ESM.zip › 11658_2019_154_MOESM1_ESM.docx]

Additional file 1

**Figure S1**. The effect of different doses of rosiglitazone on the expression of *αENaC* mRNA in mouse models of acute lung injury (ALI). The expression of *αENaC* mRNA increased gradually with increasing doses of rosiglitazone up to 4 mg/kg, which significantly increased *αENaC* mRNA expression. A further increase of rosiglitazone (8 mg/kg) did not further increase the expression of *αENaC* mRNA. The data are presented as the means ± SEM (n = 3) and analyzed with SPSS 13.0using ANOVA followed by LSD post-test for multiple comparisons. ^#^p > 0.05 vs. control group; *p < 0.05 vs. control group; ^Δ^p > 0.05 vs. RGZ 4 mg/kg group.

**Figure S2.** The effect of different doses of GW9662 on the rosiglitazone-increased expression of *αENaC* mRNA in mouse models of ALI. The expression of *αENaC* mRNA decreased gradually with increasing doses of GW9662 up to 1 mg/kg, which significantly decreased *αENaC* mRNA expression compared with the control group. A further increase of GW9662 (2 mg/kg) did not further decrease the expression of *αENaC* mRNA. The data are presented as the means ± SEM (n = 3) and analyzed with SPSS 13.0using ANOVA followed by LSD post-test for multiple comparisons. ^#^p > 0.05 vs. control group; *p < 0.05 vs. control group; ^Δ^p > 0.05 vs. RGZ 4 mg/kg + GW9662 1 mg/kg group.

**Figure S3.** The effect of different doses of rosiglitazone on the expression of *αENaC* mRNA in alveolar epithelial cells. The expression of *αENaC* mRNA in alveolar cells increased gradually with increasing doses of rosiglitazone, up to 15 μM, which significantly increased the *αENaC* mRNA expression in alveolar epithelial cells. A further increase of rosiglitazone (20 μM) did not further increase the expression of *αENaC* mRNA. The data are presented as the means ± SEM (n = 3) and analyzed with SPSS 13.0 using ANOVA followed by LSD post-test for multiple comparisons. ^#^p > 0.05 vs. control group; *p < 0.05 vs. control group; ^Δ^p > 0.05 vs. RGZ 15 μM group.

**Figure S4.** The effect of different doses of GW9662 on the rosiglitazone-increased expression of *αENaC* mRNA in alveolar epithelial cells. The expression of *αENaC* mRNA decreased gradually when increasing the dose of GW9662 in alveolar epithelial cells up to 10 μM GW9662, which significantly decreased the *αENaC* mRNA expression compared with the control group. A further increase of GW9662 (20 μM) did not further decrease the expression of *αENaC mRNA*. The data are presented as the means ± SEM (n = 3) and analyzed with SPSS 13.0 using ANOVA followed by LSD post-test for multiple comparisons. ^#^p > 0.05 vs. control group; *p < 0.05 vs. control group; ^Δ^p > 0.05 vs. RGZ 15 μM + GW9662 10 μM group.
